# Public preferences for ecological indicators used in Everglades restoration

**DOI:** 10.1371/journal.pone.0234051

**Published:** 2020-06-18

**Authors:** G. Andrew Stainback, John H. Lai, Elizabeth F. Pienaar, Damian C. Adam, Ruscena Wiederholt, Chloe’ Vorseth

**Affiliations:** 1 The Everglades Foundation, Palmetto Bay, Florida, United States of America; 2 Food and Resource Economics Department, University of Florida, Gainesville, Florida, United States of America; 3 Department of Wildlife Ecology and Conservation, University of Florida, Gainesville, Florida, United States of America; 4 Mammal Research Institute, University of Pretoria, Pretoria, South Africa; 5 School of Forest Resources and Conservation, Gainesville, Florida, United States of America; 6 Department of Earth and Environment, Florida International University, Miami, Florida, United States of America; Chinese Academy of Sciences, CHINA

## Abstract

The Everglades is one of the largest wetland ecosystems in the world covering almost 18,000 square miles from central Florida southward to Florida Bay. Over the 20th century, efforts to drain the Everglades for agriculture and development severely damaged the ecosystem so that today roughly 50% of the historic flow of water through the Everglades has been diverted elsewhere. In an attempt to restore the Everglades, the U.S. Congress authorized the Comprehensive Everglades Restoration Plan (CERP) in 2000, expected to cost over $16 billion and to take several decades to complete. We used the results from a stated preference choice experiment (SPCE) survey of Florida households to estimate the willingness to pay for several ecological attributes related to CERP performance indicators likely to be impacted by Everglades restoration. We also used a latent class model (LCM) to explore preference heterogeneity among respondents. On average, survey respondents were willing to pay for improvements in all of the attributes included in the survey, namely increased populations of wading birds, American alligators, endangered snail kites, and spotted seatrout, and reduced polluted discharges from Lake Okeechobee to the Caloosahatchee and St. Lucie rivers. Willingness to pay was highest for reduced polluted discharges from Lake Okeechobee.

## Introduction

Ecological restoration to protect and enhance ecosystem services is increasingly recognized as important in promoting both conservation and human welfare [1–3]. For example, in 2012 the Convention on Biological Diversity articulated “ambitious but attainable goals for scaling up efforts to restore and rehabilitate degraded ecosystems and landscapes around the world” [4]. Around 10,000 governments and NGOs agreed that ecosystem restoration was important to sustainable development in the Jeju Declaration of the fourth World Congress of International Union for Conservation and Nature (IUCN) [4]. Monetizing the changes in benefits or ecosystem services resulting from ecological restoration is important to attain sustainable development for several reasons. First, the costs of restoration are generally presented in monetary terms so to compare the benefits of restoration to the investment needed, monetizing ecosystem services is necessary [5]. Second, there is often a need to prioritize different restoration projects or choose among several options in achieving a restoration goal. Considering the full range of benefits and costs associated with different restoration projects or options can help decision-makers choose the more effective or beneficial course of action. Finally, fully accounting for the benefits of restoration can illuminate how the benefits are distributed among different stakeholders [5].

However, there are multiple challenges in incorporating the monetary value of the benefits resulting from ecological restoration into decision-making. First, the lack of in-depth local data on the value of these benefits substantially hinders the use of this kind of information in decision-making [6–8]. Second, many decision-makers consider ecosystem services and their valuation “a relatively new and complex approach that needs more rigorous testing” [6,9]. Poor communication between experts (scientists) and non-experts (e.g., policy- and decision-makers, the public) around the concept of ecosystem services can also lead to reduced political support for its use [6,9]. Finally, monitoring and assessment of environmental outcomes often rely on ecological indicators based on a scientific and technical understanding of ecosystem processes [10]. However, effective decision-making regarding restoration requires both an understanding of how the public values different restoration outcomes and the ability to communicate the tradeoffs involved in decisions to policy-makers [10–12]. This study was conducted to partially address some of these challenges by estimating the value of several ecological attributes directly connected to ecological performance indicators. These indicators are being used by government agencies and policy-makers tasked with making and implementing management decisions regarding Everglades restoration. The marginal willingness to pay (WTP) estimates from this study can be explicitly linked to existing ecological models that predict the outcomes of restoration decisions to better understand the tradeoffs involved in such decisions.

**Fig 1:**
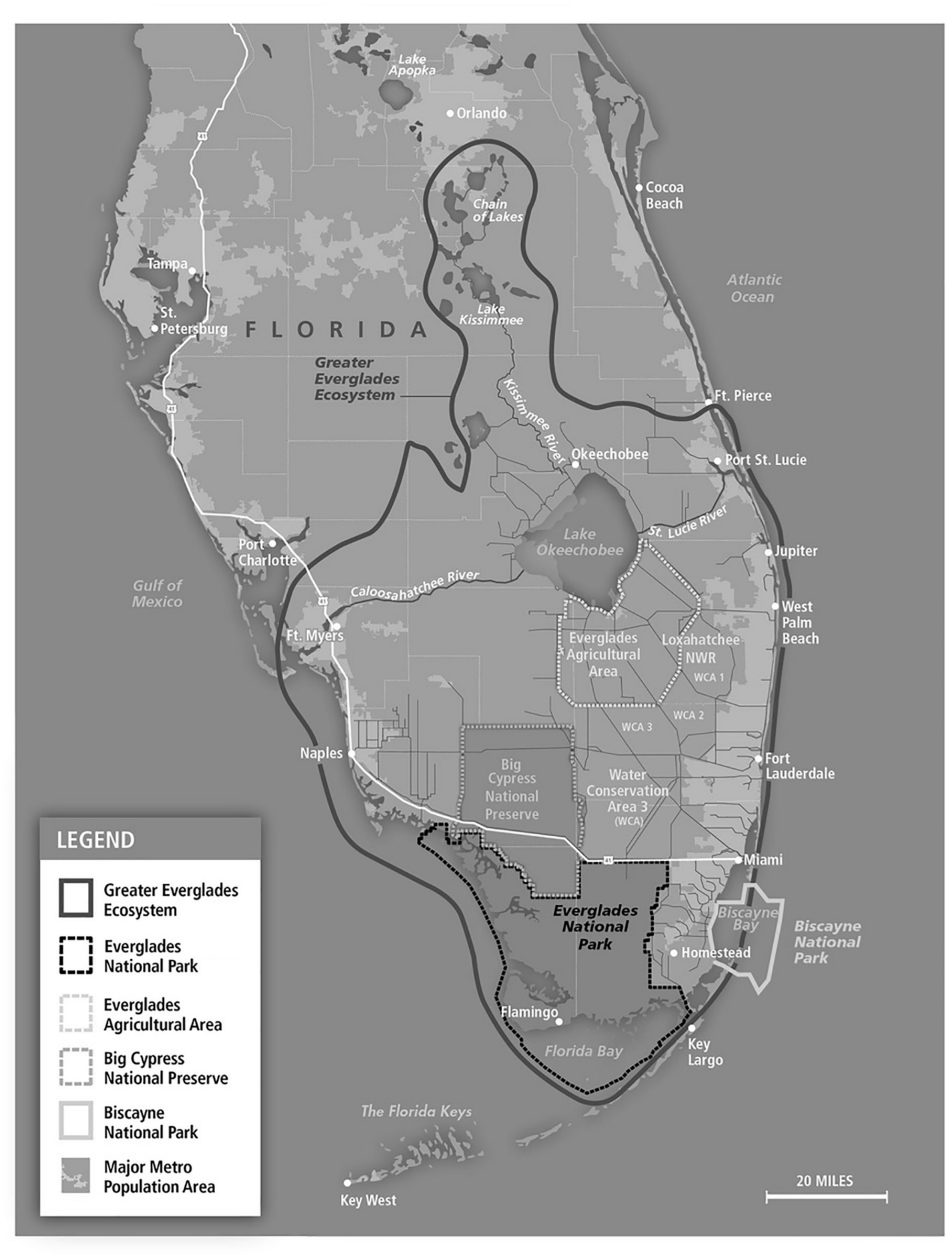
The Greater Everglades ecosystem covers almost 18,000 square miles from the Kissimmee Basin to Florida Bay – a distance of more than 200 miles. Map create by Kmusser and distributed under the Creative Commons License CC BY-SA 3.0.

Everglades restoration represents one of the world’s most substantial efforts at ecological restoration with an expected cost of over $16 billion. The Greater Everglades ecosystem is one of the world’s largest wetland ecosystems, covering almost 18,000 square miles from the Kissimmee Basin, just south of Orlando, southward to Florida Bay and the Florida Keys–a distance of more than 200 miles (Fig 1). Its location at the transition between the sub-tropical and tropical climatic zones makes it one of the world’s most unique natural areas. Historically, the ecosystem was characterized by a continuous flow of clean freshwater down the Kissimmee River Basin, through Lake Okeechobee, and across the southern Everglades before emptying into Florida Bay and the Gulf of Mexico near the Florida Keys. However, over the 20th century, efforts to drain the Everglades for agriculture and development severely damaged the ecosystem, so that today roughly 50% of the historic flow of water through the Everglades has been diverted. Currently, the Everglades is about half its original size and the southern Everglades is disconnected from Lake Okeechobee and the Kissimmee Basin. This ecological disconnection has resulted in the loss of approximately 1.7 billion gallons of freshwater each day into the Gulf of Mexico and the Atlantic Ocean, declining water quality, and saltwater intrusion into groundwater resources owing to decreased hydraulic pressure [13]. Degradation of the Everglades has also resulted in loss of biodiversity and reduced populations of native plant and animal species [13,14].

In an attempt to restore the Everglades, Congress authorized the Comprehensive Everglades Restoration Plan (CERP) in 2000. CERP contains dozens of broadly defined project components designed to work together to allow water managers to clean the water, reduce the harmful release of water to the east and west, and re-route water flow back to the south (ultimately to Florida Bay and the Florida Keys). These projects would ultimately help restore the remaining Everglades and improve habitats for native species [14].

Despite the ecological significance of the Everglades, very few valuation studies [8,13,15]. have been conducted to inform decision-making and policy regarding Everglades restoration. Owing to the range of ecosystem services associated with Everglades restoration, none of these studies provided a comprehensive valuation of Everglades restoration. For example, Milon and Scrogin [15] estimated WTP for both functional (spatial and temporal variations in water levels) and structural (changes in broadly defined populations of wetland, dryland and estuarine-dependent animal species) attributes of Everglades restoration. Latent class analysis demonstrated considerable heterogeneity in Florida residents’ preferences for Everglades restoration, and that Florida residents place higher value on structural attributes of Everglades restoration, in particular increased populations of wetland-dependent species [15]. These results were verified by Seeteram et al. [13], who found higher levels of support across Florida residents for species restoration scenarios over hydrological restoration scenarios, in all likelihood because the public does not readily understand the intricacies and benefits of hydrological restoration of the Everglades [15]. Florida residents would be more likely to support Everglades restoration if the links to species protection were highlighted [15], which is consistent with research showing that people place higher value on biodiversity conservation and the continued existence of endangered species than climate-change mitigation from altered land-use planning [16]. Finally, Richardson et al. [8] found that $1.8 billion in ecosystem services (climate regulation, improved commercial and recreational fishery harvests, increased recreational access to the Everglades, improved water quality, increased water supply) would be secured through the implementation of the Central Everglades Planning Project (CEPP), a portion of CERP concerned with restoring the central Everglades. This conservative estimate was generated using existing market data, benefits transfer methods, and cost-based approaches [8]. Each of these previous studies noted the need for further valuation studies to complement their findings and inform decision-making.

To build on the above valuation studies, we used data from a stated preference choice experiment (SPCE) survey to estimate how people would value possible changes that would result from Everglades restoration to wading bird and American alligator populations in Everglades National Park, the population of the endangered snail kite in the greater Everglades Ecosystem, spotted seatrout in Florida Bay, and polluted discharges from Lake Okeechobee to the Caloosahatchee and St. Lucie rivers during wet periods. All of these attributes are directly connected to specific CERP performance indicators developed by RECOVER (Restoration Coordination & Verification), a multiple-agency team composed of scientists and other experts from both federal and state agencies, who are tasked with assessing the progress of Everglades restoration and the expected responses of the ecosystem to alternative restoration strategies. Furthermore, there are existing peer-reviewed ecological and hydrological models that predict how each of these attributes will respond to Everglades restoration [17–20], which would allow WTP estimates generated by our study to be linked to specific ecological models that can be used to estimate marginal changes in benefits as a result of restoration choices in the future. Ours is also the first study regarding WTP for reduced polluted discharges from Lake Okeechobee to the St. Lucie and Caloosahatchee rivers–an important restoration benefit that has received much media and political attention due to polluted discharges being linked to recent damaging algae blooms.

In addition, as part of the survey, respondents were queried about their environmental attitudes using the New Ecological Paradigm scale and other attitudinal questions specific to Everglades restoration. A latent class model was used to explore how preferences for Everglades restoration varied with the sociodemographic characteristics of respondents and their attitudes toward the environment and restoration.

## Methodology

### Survey and choice experiment design

The data for this study was obtained from a survey conducted by the Everglades Foundation (a non-profit private foundation) in 2017 for the purpose of gathering information for its policy and communications strategy. The survey was administered by Qualtrics, a private market research firm, to a panel of adult respondents who volunteered in exchange for incentives (e.g. cash, gift cards, airline miles). All participants in panels used by Qualtrics must consent to being a part of the panel by submitting a form requesting to participate in market research. Potential respondents were sent an email invitation to complete the survey and were given information regarding the purpose of the survey, its basic content, and the estimated time needed to complete it. Participants could choose to end their participation at any time. The Everglades Foundation is not a Federalwide Assurance Institution (FWA) under U.S. Department of Health and Humans Services regulations nor were any federal government funds used to conduct the survey. Thus, U.S. federal regulations requiring Institutional Review Board (IRB) approval for research involving human subjects did not apply to administration of the survey (see 45 C.F.R. § 46, 2016). The survey and its administration adhered to the ethical and other standards set forth in the ICC/ESOMAR International Code on Market, Opinion and Social Research and Data Analytics.

The survey was comprised of five parts: (1) an introduction that explained the purpose of the survey and asked respondents to provide their sociodemographic characteristics (e.g., age, gender, race/ethnicity, and income); (2) a brief description of the Everglades, how it has changed from its original state, and current conservation efforts embodied in CERP; (3) a description of the ecological and cost attributes included in the choice experiment; (4) the SPCE questions and follow-up questions to test for biased or strategic responses; and (5) questions related to respondents’ political ideology and environmental attitudes. Environmental attitudes were measured using the New Ecological Paradigm scale [21,22] and other questions specific to Everglades restoration. Respondents were presented with a series of six choice scenarios with each choice scenario containing two restoration options (Choice A and Choice B) with differing levels of improvement in ecological attributes and an associated annual cost, and an opt-out option labeled Choice C with no ecological restoration and zero cost, which helps mitigate potential bias [23]. After making their choices, respondents were presented with a series of questions asking whether they thought cost should be a factor in ecological restoration of the Everglades, if they found the choice experiment questions difficult to answer, if they were concerned about the government’s ability to manage ecological restoration programs, and whether they thought it was fair for them to have to pay for restoration of the Everglades.

A set of potential attributes for the choice experiment were chosen based on a comprehensive literature review [13,15,17–20,24–36] and informal interviews with several hydrologists and ecologists familiar with the Everglades ecosystem who are engaged in research and/or management regarding this restoration effort. Attributes were evaluated based on their connection to CERP approved indicators, whether there were existing research or models published in the peer-reviewed literature that could be used to quantify how the attributes would respond to various restoration scenarios, and whether they could be easily understood by Florida residents. The attributes included in the survey were wading bird populations in Everglades National Park, the American alligator population in Everglades National Park, the population of the endangered snail kite in the greater Everglades Ecosystem (see Fig 1), the population of spotted seatrout in Florida Bay, and the amount of polluted discharges from Lake Okeechobee to the Caloosahatchee and St. Lucie rivers during wet periods.

Increased alligator populations are considered to be a system-wide performance metric for restoration of the hydrology in critical areas of the Everglades ecosystem [36]. Habitat suitability models for the American alligator—*Alligator mississippiensis* have been developed over the past several decades [20,26,28,29,31]. Wading birds are considered a performance metric for the resumption of the flow of fresh water through the southern Everglades [33]. A species distribution, foraging and reproduction model has been developed for wading birds including Great egrets (*Ardea allea*), white ibises (*Eudocimus albus*), and wood storks (*Mycteria Americana*) [17]. Snail kites rely almost exclusively on one prey item, the apple snail (*Pomacea paludosa*) [25]. Everglade snail kite populations are considered to be an important indicator of water quality in wetland ecosystems throughout the Greater Everglades [32]. A spatially explicit apple snail population model [18] has been developed that can be used to predict how restoration will impact Everglade snail kite populations. Florida Bay supports recreational fisheries for various species including spotted seatrout (*Cynoscion nebulous*) [27]. Spotted seatrout populations are considered a key indicator for the restoration of freshwater sheet flow into Florida Bay that are necessary for the alleviation of hypersaline conditions [35]. Spotted seatrout populations’ responses to salinity and water temperature can be predicted from an existing habitat suitability model [19]. Finally, during especially wet periods, water is discharged from Lake Okeechobee to the St. Lucie River (to the east) and the Caloosahatchee River (to the west). These polluted discharges lead to periodic toxic algae blooms, seagrass and oyster die-offs, and other detrimental ecological impacts [24,30]. Improvement in the ecological conditions of the St. Lucie and Caloosahatchee rivers and estuaries are considered a measure of successful water management in Lake Okeechobee as part of Everglades restoration [34]. The descriptions of these attributes that were presented to survey respondents are shown Table 1. Levels for the ecological attributes encompass realistic expected ecological responses to restoration as determined by a literature review and discussions with experts familiar with Everglades restoration and ecology. Table 1 also describes the levels of each of the attributes.

**Table 1:** Description of attributes and their levels presented to respondents in the choice experiment section of the survey.

Attribute	Description	Levels
Wading birds in Everglades National Park	The Everglades provides critical habitat for wading birds such as wood storks, great egrets, and white ibis. Wading birds also are important indicators of ecosystem health. The success of wading bird populations depends on how water flows through the Everglades. Over the last century, wading bird populations have declined as much as 90% partly due to the loss of suitable habitat and unnatural fluctuations in water levels. Restoring more natural water flows through the Everglades is expected to increase wading bird habitat and populations.	10%, 50% and 75% increase above current populations
American alligators in Everglades National Park	By digging holes and other activities, alligators help retain water in the dry season and form important habitat for other species. Alligators are also important indicators of ecosystem health for the Everglades. Alligators are very sensitive to water conditions that affect their food sources and ability to reproduce. By restoring the timing and extent of water flowing through the Everglades to more natural conditions, Everglades restoration is expected to increase the available habitat for alligators and increase their populations.	10%, 50% and 75% increase above current population
Endangered Everglade snail kite in the Greater Everglades	The Everglade snail kite is a hawk-like bird that depends on apple snails as its sole food source. They are found in central and south Florida and listed as endangered under the Endangered Species Act. Currently the Everglades snail kite population is estimated to be less than 1,500 individuals. By restoring the natural timing and extent of water flowing through the Everglades, restoration is expected to increase apple snail populations and, ultimately, Everglade snail kite populations.	10%, 50% and 75% increase above current population
Spotted seatrout in Florida Bay, Everglades National Park	Spotted seatrout is an important recreational fish species in Florida Bay, a part of Everglades National Park. Because they spend their entire lifecycle in the bay, spotted seatrout are an excellent indicator of the health of seagrass beds and of Florida Bay in general. Past modifications to the Everglades severely reduced the amount of fresh water flowing through the Everglades and reaching Florida Bay. As a result, Florida Bay suffers periodic hyper-salinity events that can lead to seagrass die-offs and reduce the population of spotted seatrout in the Bay. Restoration would help reestablish historic freshwater flows to Florida Bay and increase the population of spotted seatrout.	10%, 50% and 75% increase above current population
Reduction of polluted water discharges to St. Lucie and Caloosahatchee Rivers	During especially wet periods, water is discharged from Lake Okeechobee to the St. Lucie River (to the east) and the Caloosahatchee River (to the west). These polluted discharges can lead to toxic algae blooms, seagrass and oyster die-offs, and negative consequences for property values and public health in nearby coastal communities. Everglades restoration will allow more water to be cleaned and then re-directed south of Lake Okeechobee back to the Everglades during wet periods instead of being discharged to the Caloosahatchee and St. Lucie rivers and estuaries thereby reducing the number of algae blooms and other negative consequences in these areas.	10%, 50% and 75% reduction in occurrence relative to current conditions
Annual cost per household	One possible way of paying for restoration of the Everglades would be to charge an additional utility tax to all Florida households. The state of Florida levies utility taxes for water, gas, and electricity. Virtually all Florida households pay these taxes.	$50, $75, $100 per year

The payment vehicle used for the cost attribute was a tax on utilities. This vehicle was used because nearly all Florida households would have to pay a utility tax regardless of whether they owned or rented their place of residence and because this payment vehicle had been successfully used in previous surveys of Florida residents regarding Everglades restoration [13,15]. The payment levels were based on past surveys of people’s WTP for ecological benefits in general and Everglades restoration specifically, and were set at levels that would generate sufficient revenues to cover the expected costs of restoration. [15,37,38].

**Fig 2:**
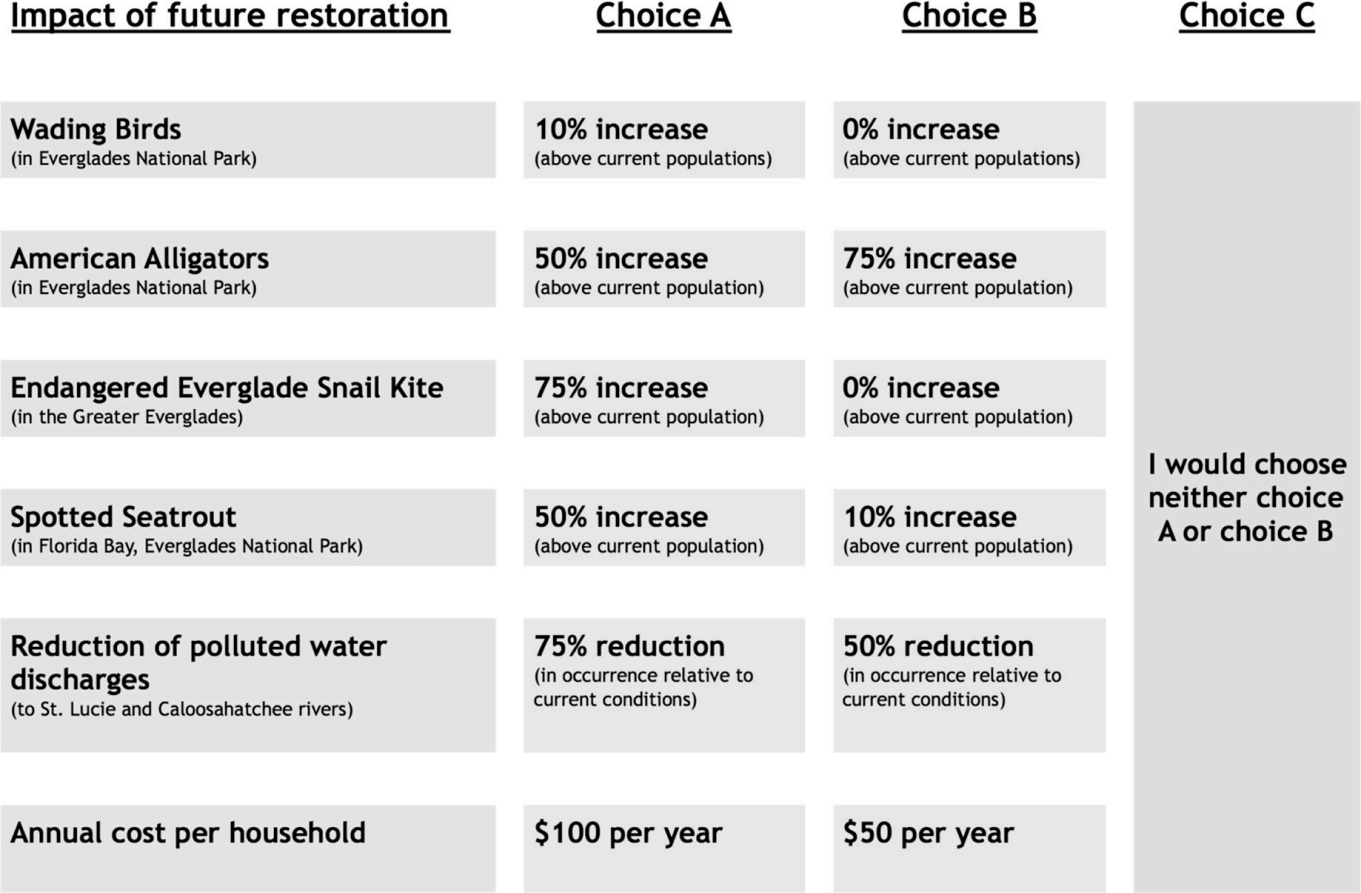
Example choice faced by survey respondent.

Owing to the number of attributes and attribute levels, a full factorial design that would include all possible combinations of attribute levels was not feasible. Alternatively, several design criteria can be used to generate an experimental design able to estimate a large number of parameters efficiently while keeping the survey short enough that respondents would be likely to complete it. These designs vary mainly in terms of orthogonality and efficiency. The software SAS [39] was used to create a D-efficient experimental design following the method outlined by Mitchell [40]. The design used in the survey consisted of eight blocks with six choice scenarios each for a total of 48 choice scenarios. Respondents were randomly assigned to one of the eight blocks. Fig 2 depicts an example choice scenario.

The survey was iteratively tested and refined based on feedback from experts in Everglades ecology and choice experiments, then pretested on a sample of 100 representative Florida residents. The survey was fully implemented (n = 2,000) as a web-based survey to a balanced panel of Florida residents in December 2017. No substantive changes were made to the survey following the pretest, resulting in a usable sample size of 2,100.

### Econometric specification and analysis

Discrete choice experiments are grounded in the random utility model (RUM) [41–44], if alternative *j* is chosen out of a set of alternatives *i = 1*, *…*., *j*, *…J* by respondent *n* in a particular choice situation *t*, then their indirect utility function can be represented as:

$$U_{njt}=\beta X_{njt}+\mu_{njt}$$ (1)

where ***X***_***njt***_ is a vector of attributes associated with alternative *j* and ***β*** is a vector of coefficients associated with the attributes of alternative *j* and characteristics of respondent *n*. The term *μ*_*njt*_ represents the random (or unobservable) component of the utility of alternative *j* for respondent *n*.

Based on the random (from perspective of the researcher) component of utility, the probability of alternative *j* being chosen over another alternative *s* in choice situation *t* can be represented as a probability function where the probability of respondent *n* choosing option *j* assuming *j ≠ s* can be represented as Eq 2 [41,44]:

$$Prob_{njt}=Prob(U_{njt}>U_{nst})=\left[(\{\;\beta X_{njt}+\mu_{njt})>(\beta X_{nst}+\mu_{nst})\right]$$ (2)

If the error terms in the above equations are independently and identically distributed and have a type I extreme value distribution, Eq (2) can be estimated using a conditional logit function [26,41]:

$$Prob_{njt}=\frac{e^{\beta X_{njt}}}{\sum_{i=1}^{J}e^{\beta X_{nit}}}$$ (3)

The dependent variable in Eq (3) is the probability that a respondent will choose a specific alternative in a choice set. The conditional logit function has been widely used to model choice behavior in discrete choice experiments [41,43–45]. However, the conditional logit model makes three limiting assumptions. It assumes homogeneous preferences across individuals, independence of irrelevant alternatives, and no correlation of unobserved factors over time [46]. These assumptions are often violated in practice; thus researchers have increasingly turned to random parameters or mixed logit models to estimate WTP for improvements in environmental quality. The random parameter logit model relaxes all three of the above assumptions [41,43] and can be represented as follows:

$$Prob_{njt}=\frac{e^{\beta_{n}X_{njt}}}{\sum_{i=1}^{J}e^{\beta_{n}X_{nit}}}$$ (4)

where **β**_***n***_ represents a vector of coefficients associated with the attributes of *j* and characteristics of respondent *n*. In the random parameter logit model at least some of the attribute coefficients are assumed to be random and thus vary across respondents [43,47].

As the dependent variable in Eqs (3) and (4) is the probability that a respondent will choose a specific alternative in a choice set, the estimated coefficients of the independent variables estimate how that particular variable will influence the probability that an alternative is chosen. Because cost was one of the attributes, the WTP for the other attributes can be calculated by taking the negative of the ratio of the estimated coefficient of the attribute of interest, **β**_***k***_, and the cost attribute as shown in Eq (5) [43]:

$${WTP}_{k}=-\frac{\beta_{k}}{\beta_{cost}}$$ (5)

When using the random parameter logit model to account for preference heterogeneity, a priori assumptions must be made regarding the distribution of the coefficients. Usually a normal or log-normal distribution is assumed. Thus, the calculation of WTP involves the ratio of two distributions where the denominator (the coefficient of the cost attribute, which is often assumed to be lognormally distributed) can typically take on values close to zero, resulting in the derived distribution of WTP being exceedingly large and unrealistic at the upper end of the distribution. This skewed distribution can bias the mean and variance of the WTP estimates [48–50]. An alternative to estimating WTP in preference space is to reparametrize the model so that the parameters in the model are marginal WTP for each attribute [48–50], i.e. the model is estimated in WTP space. Some empirical evidence suggest that models estimated in WTP space produce more reasonable distributions for WTP estimates [50]. To test the robustness of our estimates we estimated WTP using the conditional logit model, the random parameter logit model in preference space, and the random parameter logit model in WTP space. The alternative specific constant was labeled ‘optout’ and coded as 1 for the optout option and 0 for both of the restoration alternatives.

To better understand heterogeneity in preferences and the influence of respondent characteristics on respondent choices we used a latent class model where respondents were segregated into classes or segments based on their preference, attitudes, and sociodemographic characteristics. The important distinction of the latent class model relative to the random parameter model is that the vector of attribute coefficients for respondents in a class are the same (i.e., respondents within a class exhibit homogeneous preferences), but estimated coefficients vary between classes (i.e. different classes are heterogeneous in preferences) [15,41,51]. Following Pienaar et al and Milon and Scrogin [15,51], the latent class model can be represented as follows:

$${Prob}_{njt|K}=\sum_{k=1}^{K}\left[\frac{e^{\alpha \gamma_{kn}}}{\sum_{k=1}^{K}e^{\alpha \gamma_{kn}}}][\frac{e^{\beta_{k}X_{njt}}}{\sum_{i=1}^{J}e^{\beta_{k}X_{nit}}}\right]$$ (6)

Where **α** is vector of parameters and **γ** is a vector of sociodemographic, political ideology, and environmental attitude variables that determine class membership. Eq (6) represents the probability of individual *n* choosing alternative *j* in choice situation *t* conditional on individual *n* belonging to class *k*. WTP is calculated the same way as in Eq (5). The latent class model with the number classes that best explained respondent heterogeneity while minimizing the risk of overfitting was determined by using the Consistent Akaike Information Criterion (CAIC) and the Bayesian Information Criterion (BIC) [51,52].

All regressions were performed using Stata 15.1 [53]. The built-in Stata *clogit* command was used to estimate the conditional logit model while the *mixlogit* and *mixlogitwtp* Stata commands developed by Arne Hole were used to estimate the random parameter logit models in preference space and WTP space, respectively [54,55]. Stata built-in commands and the user-written commands *lclogit2* and *lclogitml2* developed by Yoo [56] were used for the latent class analysis.

## Results

### Respondent demographics

Overall, the sample was fairly representative of the population of Florida. A detailed comparison of the sample with Florida demographics is presented in S1 Table in the supplemantry material. The sample was more educated with about 52% of the sample with a bachelor’s degree or higher, compared to around 28% of the population. The sample also had a higher income with a median household income of $70,000 per year, compared to around $49,000 for Florida’s population. The survey did not ask respondents about their political party affiliation, but they were asked to identify themselves as liberal or conservative on a 7-point Likert scale, with one being the most conservative and seven being the most liberal. Even though it is recognized that the political party identification is imperfectly correlated with liberal/conservative ideology [57,58] if those self-identifying from one to three are classified as conservative, those identifying as a 4 as independent, and those identifying at 5–7 as liberal, then these classifications roughly aligned with the population identification of Republican, Independent, and Democrat respectively. Other demographic variables were similar between the sample and the population of Florida.

### Logit model results

A description of the variables used in the conditional logit and the random parameter logit models are shown in Table 2. The results from the conditional logit model, the random parameter model in preference space, and the random parameter model in WTP space were broadly consistent with each other. For all three models, the estimated coefficients had the theoretically expected signs. The ecological attributes (increased populations of wading birds, American alligators, Everglades snail kites, spotted seatrout, and reduced polluted discharges from Lake Okeechobee) all had positive coefficients, meaning that their presence increased the probability of a restoration option being chosen, all else being equal. The estimated coefficient for the cost attribute was negative, meaning that all else being equal, the higher the cost the less likely it would be that a respondent would choose a restoration option. The estimated coefficient for the optout variable was negative, indicating that, overall, respondents preferred restoration of the Everglades over the status quo.

**Table 2:** Description of variables used in regression models.

Attribute	Description
Restoration	Constant for alternatives. 0 = opt out alternative, 1 equals one of the restoration alternatives.
bird10	Increase wading bird populations in Everglades National Park by 10% above current levels
bird50	Increase wading bird populations in Everglades National Park by 50% above current levels
bird75	Increase wading bird populations in Everglades National Park by 75% above current levels
gator10	Increase in American alligator populations in Everglades National Park by 10% above current levels
gator50	Increase in American alligator populations in Everglades National Park by 50% above current levels
gator75	Increase in American alligator populations in Everglades National Park by 75% above current levels
snail10	Increase in Everglades snail kite population in the greater Everglades ecosystem by 10% above current levels
snail50	Increase in Everglades snail kite population in the greater Everglades ecosystem by 50% above current levels
snail75	Increase in Everglades snail kite population in the greater Everglades ecosystem by 75% above current levels
trout10	Increase in spotted seatrout population in Florida Bay by 10% above current levels
trout50	Increase in spotted seatrout population in Florida Bay by 50% above current levels
trout75	Increase in spotted seatrout population in Florida Bay by 75% above current levels
water10	Reduction of polluted water discharges to St. Lucie and Caloosahatchee Rivers by 10% from current levels
water50	Reduction of polluted water discharges to St. Lucie and Caloosahatchee Rivers by 50% from current levels
water75	Reduction of polluted water discharges to St. Lucie and Caloosahatchee Rivers by 75% from current levels
cost	Annual cost per household ($)

The results for the random parameter logit models in both preference space and WTP space are shown in Table 3. Because all models produced similar results, the results from theconditional logit model are presented in S2 Table in the supplementary material. With the exception of a 50% increase in the American alligator population, coefficients estimated in preference space were significant at the 5% level. For the model estimated in WTP space, the coefficients for all attributes were significant at the 1% level. Both random parameter logit models indicated that respondents’ preferences for ecological restoration were heterogeneous, although the models were not entirely consistent in terms of which standard deviation coefficients were statistically significant. The magnitude of WTP estimates was similar across the conditional logit and random parameter logit models, with respondents showing the greatest WTP for reduced discharges of polluted water from Lake Okeechobee and the least WTP for increased American alligator populations in Everglades National Park. Although the WTP estimates were similar, empirical evidence suggests that estimating the random parameter logit model in WTP space instead of preference space produces more reasonable willingness to pay estimates [48,50]. That seems likely the case here as the WTP estimates from the random parameter models estimated in WTP space were all significant at the 1% level.

**Table 3:** Random parameter logit results in preference space and WTP space.

	Regression in Preference Space			Regression in WTP Space	
	Coefficient	Std. Error	WTP	Coefficient (WTP)	Std. Error
**Mean**					
Optout	-4.499***	0.142		-270.46***	11.846
bird10	0.412***	0.033	$31.83	$19.97***	3.597
bird50	0.694***	0.028	$53.67	$47.94***	3.911
bird75	0.696***	0.026	$53.79	$44.75***	3.688
gator10	0.153**	0.032	$11.80	$17.31***	3.709
gator50	0.060	0.029	$4.65	$12.65***	3.299
gator75	0.132**	0.028	$10.22	$13.32***	3.565
snail10	0.173***	0.032	$13.40	$16.72***	3.587
snail50	0.564***	0.033	$43.62	$45.10***	4.594
snail75	0.531***	0.027	$41.01	$42.09***	3.391
trout10	0.454***	0.030	$35.08	$30.66***	3.448
trout50	0.449***	0.034	$34.69	$36.60***	3.828
trout75	0.584***	0.024	$45.11	$41.99***	3.624
water10	0.652***	0.034	$50.35	$40.84***	4.427
water50	1.108***	0.032	$85.63	$76.28***	5.081
water75	1.390***	0.032	$107.41	$95.80***	3.597
cost	-4.348***	0.101		-3.983***	0.081
**Standard Deviation**					
Optout	1.978***	0.143		214.082***	12.770
bird10	0.133	0.124		18.226***	5.727
bird50	0.315*	0.092		25.282***	2.724
bird75	0.067	0.061		4.142	4.071
gator10	0.646***	0.060		30.948***	5.086
gator50	0.018	0.100		7.789	3.604
gator75	0.546***	0.055		33.836***	4.819
snail10	0.455***	0.081		30.724***	4.254
snail50	0.194	0.096		1.188	4.332
snail75	0.436***	0.054		22.599***	6.814
trout10	0.410	0.085		32.778***	5.322
trout50	0.867***	0.062		60.476***	3.823
trout75	0.100	0.080		3.896	4.503
water10	0.505***	0.091		2.358	8.676
water50	0.660***	0.062		10.527	5.727
water75	1.134***	0.037		61.687***	2.724
cost	1.633***	0.106		1.197***	0.107
Log Likelihood	-10,750.681			-10,758.264	
N	37,797			37,797	
LR chi^2^(17)	4,579.51			3,160.87	
Prob>chi^2^	0.0000			0.0000	
Halton draws	500			500	

*** 1% significance level

** 5% significance level

* 10% significance level

Coefficients for attributes, except cost, in WTP space represent willingness to pay estimates.

Willingness to pay for an increase in the American alligator and spotted seatrout populations remained approximately the same as the population level increased, with mean WTP at each population increase being well within the 95% confidence interval of the other levels. Respondents were willing to pay for a relatively small increase in these species populations but were relatively indifferent to further increases beyond an initial population increase of 10 percent. Respondents’ WTP for increases in the populations of wading birds and the Everglade snail kite increased when the expected increase in population size transitioned from 10% to 50%, but further population increases did not result in much change in WTP. Respondents’ WTP for reduced discharges of polluted water from Lake Okeechobee to the St. Lucie and Caloosahatchee rivers increased in a roughly linear fashion as reductions in polluted discharges increased.

### Latent class model results

To better understand the nature of the preference heterogeneity among survey respondents, a latent class model specification was used where respondents were divided into distinct classes based on their responses to the New Ecological Paradigm (NEP) scale [22], other attitudinal questions, and respondent socioeconomic characteristics (see Table 4). The NEP scale has been widely used to measure environmental attitudes of survey respondents [59]. The scale consists of 15 statements where the respondent is asked to indicate their level of agreement or disagreement for each statement on a 5-point Likert scale (see Table 5). Eight of the 15 statements express sentiments that endorse an eco-centric or ecological world view, while the other 7 statements express sentiments that align with a more anthropocentric or “Dominant Social Paradigm” world view [22]. Previous research has shown that responses to the 15 NEP questions measure a latent attitude or worldview concerning how humans relate to the natural world [21,22,59].

**Table 4:** The sociodemographic and attitudinal questions used in the latent class model.

Variable	Description
female	Gender of respondent *(1 if female)*
Race*	Race of the respondent
*black*	Black or African American (1 if Black or African American)
*0ther_race*	Other (1 if neither *Black or African American* or *White*
hispanic	Spanish, Hispanic, or Latino *(1 if yes)*
age	Age of respondent
college	Respondent has a college degree
income	Income of respondent
politics	Political views of respondent *(7-point Likert scale with -3 being extremely liberal and 3 extremely conservative)*
nep	Composite score from 15 to 75 based on responses to the new ecological paradigm questions. A higher score indicates an attitude more aligned with the New Ecological Paradigm. A lower score indicates an attitude more aligned with the Dominant Social Paradigm
nocost	Cost should not be a factor in restoration of the Everglades *(5-point Likert scale of strongly disagree [–2] to strongly agree [2])*
nogovt	I am concerned that the government cannot manage programs effectively. *(5-point Likert scale of strongly disagree [–2] to strongly agree [2])*
nopay	I should not have to pay additional fees or taxes for Everglades restoration. *(5-point Likert scale of strongly disagree [–2] to strongly agree [2])*

*Respondents who self-identified as white were coded as the reference category

**Table 5:** Questions composing the new ecological paradigm scale. Agreement with unshaded questions indicate a “eco-centric” view, while agreement with shaded questions indicate a “anthropocentric” world view.

	Strongly Agree	Mildly Agree	Unsure	Mildly Disagree	Strongly Disagree
We are approaching the limit of the number of people the earth can support.					
Humans have the right to modify the natural environment to suit their needs.					
When humans interfere with nature it often produces disastrous consequences.					
Human ingenuity will ensure that we do NOT make the earth unlivable.					
Humans are severely abusing the environment.					
The earth has plenty of natural resources if we just learn how to develop them.					
Plants and animals have as much right as humans to exist.					
The balance of nature is strong enough to cope with the impacts of modern industrial nations.					
Despite our special abilities, humans are still subject to the laws of nature.					
The so-called "ecological crisis" facing humankind has been greatly exaggerated.					
The earth is like a spaceship with very limited room and resources.					
Humans were meant to rule over the rest of nature.					
The balance of nature is very delicate and easily upset.					
Humans will eventually learn enough about how nature works to be able to control it.					
If things continue on their present course we will soon experience a major ecological catastrophe.					

**Fig 3:**
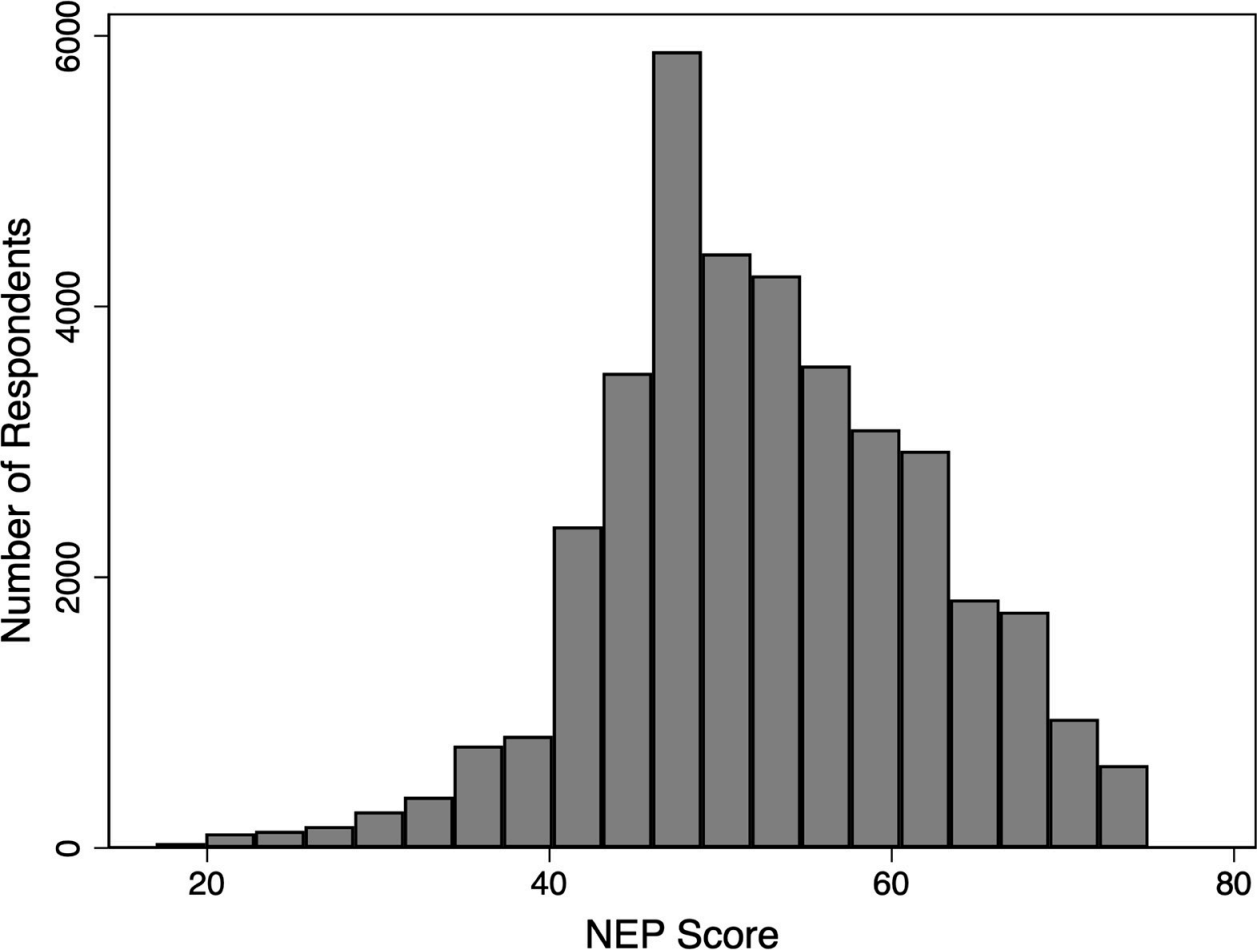
Histogram of NEP scores. Statements expressing an “anthropocentric” view were reverse coded. Scores range from 15 to 75 with higher scores indicating a more “eco-centric” attitude toward nature.

The responses to the NEP scale statements can be aggregated into a single score ranging from 15 to 75 to indicate variation in respondents’ underlying attitudes, with a higher score indicating a more eco-centric attitude. Fig 3 shows a histogram of aggregate NEP scores from the survey respondents displaying an approximately normal distribution with a slight skew toward an eco-centric world view. This result is consistent with other studies using the NEP scale [22,60–62]. The interitem correlations, which measure if individual questions on a questionnaire, are correlated) and Cronbach’s alpha, which measure the internal reliability of survey questions, are shown in Table 6. The average interitem correlation was 0.329 on the test scale which is within the ideal range of 0.15 to 0.50 and the average Cronbach’s alpha was 0.820 which shows a good level of internal reliability. The Cronbach’s alpha score on each individual item indicates how much the average Cronbach’s alpha would decrease if that item was removed. As can be seen in Table 6, removing any of the items would decrease the average, indicating that no item should be removed from the scale to improve reliability.

**Table 6:** Interitem correlations and Cronbach’s alpha for NEP questions.

Test scale = mean(unstandardized items)						
Item	Obs.	Sign	item-test correlation	item-rest correlation	average interitem covariance	alpha
nep1	2100	+	0.457	0.344	0.337	0.816
nep2	2100	-	0.578	0.473	0.319	0.807
nep3	2100	+	0.499	0.406	0.336	0.811
nep4	2100	-	0.417	0.313	0.345	0.817
nep5	2100	+	0.615	0.535	0.322	0.803
nep6	2100	-	0.444	0.336	0.340	0.816
nep7	2100	+	0.494	0.406	0.338	0.811
nep8	2100	-	0.632	0.536	0.311	0.802
nep9	2100	+	0.401	0.318	0.351	0.816
nep10	2100	-	0.702	0.616	0.299	0.795
nep11	2100	+	0.468	0.356	0.336	0.815
nep12	2100	-	0.587	0.479	0.316	0.806
nep13	2100	+	0.529	0.446	0.335	0.809
nep14	2100	-	0.494	0.389	0.334	0.813
nep15	2100	+	0.644	0.562	0.316	0.801
*Test scale*					0.329	0.820

Test scale = mean(unstandardized items)

Results from the latent class model confirm that there is preference heterogeneity among the survey respondents. According to the Consistent Akaike Information Criterion (CAIC), a model with four classes provides the best fit, and according to the Bayesian Information Criterion (BIC), a model with five classes provides the best fit while minimizing the risk of overfitting. To use the simplest model and ensure that the model did not overfit the data, we used a four-class model. Results of the latent class model are presented in Table 7 and Table 8 presents the willingness to pay estimates calculated using Eq 5.

**Table 7:** Results from the latent class model.

	Class 1		Class 2		Class 3		Class 4	
	*Coefficient*	*Std*. *Error*	*Coefficient*	*Std*. *Error*	*Coefficient*	*Std*. *Error*	*Coefficient*	*Std*. *Error*
*optout*	3.911***	1.042	-7.189***	0.618	-2.982***	0.164	-2.982***	0.164
*bird10*	-0.176	0.242	0.396***	0.102	0.051	0.043	0.051***	0.043
*bird50*	-0.191	0.245	0.691***	0.080	0.126***	0.036	0.126***	0.036
*bird75*	-0.126	0.200	0.758***	0.082	0.124***	0.034	0.124***	0.034
*gator10*	0.010	0.253	0.274	0.117	0.021	0.039	0.021**	0.039
*gator50*	-0.484	0.320	0.273**	0.097	0.030	0.040	0.030	0.040
*gator75*	-0.016	0.219	0.192*	0.102	0.069*	0.036	0.069**	0.036
*snail10*	-0.106	0.204	0.130	0.101	0.074*	0.043	0.074	0.043
*snail50*	-0.631	0.338	0.484***	0.113	0.168***	0.044	0.168***	0.044
*snail75*	-0.405	0.210	0.502***	0.101	0.166***	0.036	0.166***	0.036
*trout10*	0.044	0.290	0.481***	0.094	0.087**	0.040	0.087***	0.040
*trout50*	-0.146	0.303	0.674***	0.122	0.068	0.043	0.068***	0.043
*trout75*	-0.221	0.205	0.714***	0.095	0.124***	0.032	0.124***	0.032
*water10*	0.541	0.253	0.786***	0.110	0.074*	0.041	0.074***	0.041
*water50*	0.019	0.351	1.397***	0.126	0.107**	0.039	0.107***	0.039
*water75*	0.198	0.247	2.053***	0.161	0.081**	0.037	0.081***	0.037
*cost*	-0.017*	0.009	-0.002	0.003	-0.011***	0.001	-0.011***	0.001
*nep*	-0.078***	0.011	-0.016	0.010	-0.051***	0.010	---	---
*politics*	-0.157**	0.065	-0.137**	0.063	0.121**	0.060	---	---
*nocost*	-0.022	0.068	0.243***	0.067	0.398***	0.066	---	---
*nopay*	0.722***	0.089	-0.314***	0.071	-0.126*	0.070	---	---
*nogovt*	-0.308**	0.095	-0.093	0.090	-0.240**	0.086	---	---
*female*	-0.306	0.203	-0.269	0.186	-0.489**	0.182	---	---
*age*	0.006	0.006	-0.004	0.006	-0.033***	0.006	---	---
*income*	0.009	0.041	0.102**	0.035	-0.024	0.037	---	---
*college*	-0.167	0.198	0.428**	0.185	0.452**	0.177	---	---
*black*	-0.307	0.273	-1.178**	0.405	0.392*	0.236	---	---
*other_race*	-0.032	0.299	-0.422	0.289	0.103	0.250	---	---
*hispanic*	-0.028	0.234	0.002	0.220	0.627**	0.195	---	---
*cons*	3.343***	0.764	0.799	0.721	4.815***	0.680	---	---
*Class Share*		15.7%		23.5%		38.3%		22.5%	

Log likelihood -9860.5974

*** 1% significance level

** 5% significance level

* 10% significance level

Demographic and socioeconomic coefficients for class 4 are normalized to 0 to facilitate interpretation.

**Table 8:** Willingness to pay estimates for each class from the latent class model.

	Class 1			Class 2			Class 3			Class 4		
	Mean	95% Conf. Interval		Mean	95% Conf. Interval		Mean	95% Conf. Interval		Mean	95% Conf. Interval	
*optout*	-$10.26	-$39.65	$19.13	$191.48	-$418.48	$801.43	$4.81	-$3.31	$12.93	$14.40	$9.37	$19.42
*bird10*	-$11.09	-$42.62	$20.44	$333.85	-$699.99	$1,367.70	$11.98	$4.41	$19.56	$18.77	$13.61	$23.93
*bird50*	-$7.30	-$31.37	$16.76	$366.03	-$763.62	$1,495.69	$11.76	$4.51	$19.01	$17.30	$12.57	$22.03
*bird75*	$0.56	-$28.21	$29.33	$132.16	-$273.98	$538.30	$2.03	-$5.30	$9.35	$7.92	$3.34	$12.51
*gator10*	-$28.16	-$71.32	$14.99	$132.09	-$288.48	$552.67	$2.81	-$4.65	$10.28	$2.76	-$1.23	$6.75
*gator50*	-$0.92	-$25.70	$23.87	$92.56	-$178.58	$363.70	$6.61	-$0.20	$13.41	$4.89	$1.12	$8.65
*gator75*	-$6.17	-$29.36	$17.03	$62.77	-$124.88	$250.43	$7.07	-$1.25	$15.38	$2.01	-$3.07	$7.08
*snail10*	-$36.69	-$86.03	$12.65	$233.92	-$508.63	$976.48	$15.97	$6.59	$25.34	$12.40	$7.85	$16.95
*snail50*	-$23.54	-$53.34	$6.25	$242.55	-$486.87	$971.97	$15.82	$8.37	$23.27	$11.55	$7.80	$15.30
*snail75*	$2.58	-$31.02	$36.19	$232.61	-$473.70	$938.92	$8.32	$0.53	$16.11	$12.78	$8.37	$17.19
*trout10*	-$8.50	-$45.32	$28.32	$325.75	-$664.58	$1,316.09	$6.48	-$1.59	$14.54	$14.18	$9.57	$18.79
*trout50*	-$12.83	-$38.29	$12.63	$345.19	-$740.95	$1,431.34	$11.79	$4.93	$18.65	$14.57	$10.55	$18.59
*trout75*	$31.48	-$4.59	$67.54	$379.62	-$799.39	$1,558.63	$7.07	-$0.76	$14.91	$17.38	$11.61	$23.15
*water10*	$1.09	-$38.73	$40.90	$675.09	-$1,405.32	$2,755.50	$10.15	$2.36	$17.94	$25.84	$19.67	$32.00
*water50*	$11.51	-$17.95	$40.97	$992.19	-$2,065.59	$4,049.97	$7.70	$0.27	$15.13	$27.17	$20.38	$33.95
*water75*	-$10.26	-$39.65	$19.13	$191.48	-$418.48	$801.43	$4.81	-$3.31	$12.93	$14.40	$9.37	$19.42
*cost*	-$11.09	-$42.62	$20.44	$333.85	-$699.99	$1,367.70	$11.98	$4.41	$19.56	$18.77	$13.61	$23.93

As can be seen in Table 7, class 3 is the largest with 38.3% of the respondents while class 1 contains the least with 15.6%. Classes 2 and 4 contain 22.5% and 23.5% respectively. Class 4 was set as the base for interpretation of the coefficients of the sociodemographic and attitudinal variables used to determine class membership. All of these variables, except one, were significant at the 5% level for at least one of the classes, meaning that at least one class differed from class 4 on these variables. The only exception was the variable *other_race* for which none of the first three classes differed significantly from class four. The coefficient for the optout choice was significant for all of the classes. Classes 2, 3, and 4 had negative coefficients, indicating that respondents in these classes preferred restoration over the status quo. Class 1 had a positive coefficient for the optout, meaning the opposite for these respondents (i.e., they do not value Everglades restoration). The coefficient for the cost attribute was significant, at least at the 10% level, for classes 1, 3, and 4 indicating the higher the cost of a restoration option, the less likely the respondents in these classes would choose it. The coefficient for the cost attribute was small and not statistically significant for class 2 indicating that respondents in this class displayed attribute non-attendance for this attribute, meaning that cost was not a factor in choosing among alternatives. This resulted in a very small, non-significant coefficient for cost for respondents in this class. Since the coefficient for the cost attribute is the denominator in calculating WTP (see Eq 5), the willingness to pay estimates for the restoration attributes for these respondents were also not significant. [63–65][65]

To facilitate discussion, the attitude and demographic characteristics of each of the four classes are shown in Table 9. Class 1 skewed whiter (relative to classes 3 and 4) and more male with fewer respondents with a college degree. This class also displayed a more anthropocentric attitude based on their responses to the NEP statements and were less likely to agree that cost should not be a factor in restoration of the Everglades and more likely to agree that they should not have to pay additional fees or taxes for Everglades restoration relative to the other three classes. As indicated previously, this class had a very small or no willingness to pay for the restoration attributes and indicated a strong preference for the status quo instead of paying for restoration. Class 2 also skewed whiter and more male. However, this class had a larger number of respondents with a college degree and on average had higher income compared to the other classes. This class displayed more eco-centric views according to their responses to the NEP statements, and they self-identified as more liberal relative to other segments in terms of political ideology. However, the willingness to pay for restoration attributes for this class was not statistically significant. Classes 3 and 4 skewed more female and indicated willingness to pay for most of the restoration attributes. These classes also had higher (or more ecocentric) scores on the NEP scale relative to class 1. The difference between these two classes is that class 3 skewed more non-white and younger while class 4 had a higher willingness to pay for reduced discharges relative to class 3. Somewhat surprisingly class 3 also self-identified as more conservative than other classes in terms of political ideology.

**Table 9:** Attitude and demographic characteristics of the four classes resulting from the latent class model. Refer to Table 6 for descriptions of the variables and their coding.

Variable	Class 1	Class 2	Class 3	Class 4
nep	48.0	55.5	50.6	54.9
nocost	-0.29	0.41	0.59	0.28
nopay	1.2	-0.41	0.22	0.33
nogovt	1.01	1.01	0.86	1.11
politics	0.28	-0.9	0.57	0.19
female	0.47	0.41	0.54	0.58
age	53	55	42	51
income	$60,000- $79,000	$80,000-$99,000	$60,000- $79,000	$60,000- $79,000
college	0.42	0.67	0.51	0.46
black	0.17	0.03	0.26	0.19
other_race	0.11	0.08	0.14	0.13
white	0.73	0.89	0.6	0.68
hispanic	0.21	0.2	0.36	0.25

## Discussion and conclusions

All three model specifications, conditional logit, random parameters logit in preference space, and random parameters logit in WTP space produced results broadly consistent with each other and economic theory. Results from the random parameter models and the latent class model indicated substantial heterogeneity across respondents’ willingness to pay. Even accounting for preference heterogeneity, the results showed that, overall, survey respondents were willing to pay for Everglades restoration, specifically increased species populations and reduced water pollution that would result from restoration efforts. Respondents’ WTP were highest for actions related to ecological system integrity in the form of reduced discharges of polluted water from Lake Okeechobee to the St. Lucie and Caloosahatchee rivers and estuaries. By contrast, their WTP was lowest for increasing the population of American alligators, an iconic and charismatic species that once had endangered status under the US Endangered Species Act but was delisted in 1987 [66]. Importantly, the results suggest that Florida residents do value increasing alligator populations and are willing to pay for restoration efforts that enhance their populations, although this is a hunted game species in the state. The degree to which respondents were aware of the game species status of the American alligator was unclear.

Interestingly, the marginal WTP point estimates were monotonically increasing for improvements in water quality and spotted seatrout populations, but not for increases in the populations of snail kites, wading birds, and alligators (despite positive mean WTP estimates at all levels of population increase). These results suggest possible respondent insensitivity to scope or magnitude of the ecological improvement being valued [67]. There are several explanations for this result that are consistent with economic theory and previous studies. First, the Law of Diminishing Marginal Utility suggests that the marginal value of increases in wildlife populations should decline as population size increases [68,69]. These smaller increases in marginal value would be more difficult to detect statistically in any given survey sample [70]. The confidence intervals for the WTP estimates for different population increases for these species significantly overlap suggesting that our sample size may not have been large enough to detect small changes in the marginal WTP for increasing population sizes. Second, there is some evidence that population increases of wildlife may not be as important to people as improvements in threatened or endangered status [71]. When only considering point estimates, these results possibly suggest that there are economically optimal increases in these populations that are less than 75% for snail kites and wading birds, and less than 50% for the alligator. However, when considering confidence interval estimates, it is more precise to suggest that mean WTP for 10% improvements in these populations was positive for snail kites, wading birds, and alligators, and WTP continued to increase for snail kites and wading birds as the population size increased by 50% or 75%.

The latent class model analysis indicates that a little more than 15% of the respondents had very little or no willingness to pay for restoration and a preference for the status quo. The rest of the respondents indicated that they preferred restoration. A little more than 60% of the respondents indicated a relatively modest WTP. About 23% of the respondents indicated a very strong preference for restoration as indicated by negative coefficient with a large absolute value for the optout choice. However, this group displayed attribute non-attendance to the range of restoration costs presented in the choice experiment as indicated by a small and statistically insignificant coefficient on the cost attribute. Attribute non-attendance is a relatively common occurrence in choice experiments [63–65] and can occur for several reasons. One reason is simply that respondents ignore the attribute as a heuristic to simplify the task of making a choice. Another potential reason is that the attribute and its variation as presented in the survey did not substantially influence the utility of the presented options [65]. We cannot definitively distinguish between these two (or other) explanations for attribute non-attendance for respondents in this class for cost. However, we think that the most parsimonious interpretation for these results is that respondents in class 2 substantially preferred restoration over the status quo and that in reality had a very high willingness to pay for restoration but were not responsive to the range of restoration costs presented in the survey. This interpretation is supported by the result that respondents in this class had the strongest aversion the optout option in the choice experiment (evidenced by the optout coefficient), scored high on the eco-centric world view on the NEP statements (see Table 8), and disagreed with the survey statement “I should not have to pay additional fees or taxes for Everglades restoration” (see Table 8).

Finally, these WTP estimates were generated using robust valuation and data collection methods, but we note several limitations to this study. First, only a subset of ecological attributes were included in the survey. For instance, Everglades restoration is expected to provide substantial benefits in terms of increased water supply to southeast Florida and climate change mitigation through carbon sequestration in peat soil, seagrass beds, and mangrove forests [72–74]. The need to consider only a subset of ecological attributes is driven by a well-known cognitive limitation in the human ability to simultaneously consider large combinations of attributes [75,76]. We are, therefore, careful to interpret these results as a lower bound on Florida resident’s willingness to pay for Everglades restoration. Another limitation of this study, which also contributes to the lower bound nature of our results, is that only residents of Florida were surveyed. Everglades restoration involves a UNESCO World Heritage site that draws visitors from around the world, and enjoys broad financial and political support well beyond Florida (e.g., significant funding from the federal level). It is highly likely that the WTP for Everglades restoration is positive well beyond the political boundaries of Florida, but non-Florida WTP is not assessed here. This is an area for future work.

The results from this study can be instrumental for predictive and on-going assessments of restoration project investments. For example, a project that leads to a 10% increase in the populations of American alligators, wading birds, Everglade snail kites, and spotted seatrout, and a 50% reduction in discharges would have an aggregate WTP of around $1.3 billion per year, which is substantially more than is currently being allocated to restoration. Over the past five years, spending on CERP, which is a 50–50 partnership between the state and the Federal government, averaged $230 million dollars [14]. Given that the ecosystem services assessed here account for a small share of the total benefits of Everglades restoration, our WTP estimates offer a lower bound estimate of the economic benefits generated by this iconic ecosystem and help demonstrate the economic effectiveness of CERP. Furthermore, the heterogeneity of WTP among respondents indicate that not everyone desires restoration equally. This may be important for policy makers to consider in the context of restoration efforts, funding, and communicating with the public.

## Supporting information

S1 Table Comparison between the demographics of the survey sample respondents and the Florida population based on 2010 U.S. Census information. (DOCX)

S2 Table Regression results from conditional logit model with willingness to pay estimates. (DOCX)
