# Correction: Public preferences for ecological indicators used in Everglades restoration

**DOI:** 10.1371/journal.pone.0244398

**Published:** 2020-12-16

**Authors:** G. Andrew Stainback, John H. Lai, Elizabeth F. Pienaar, Damian C. Adams, Ruscena Wiederholt, Chloe’ Vorseth

The fourth author's name is spelled incorrectly. The correct name is: Damian C. Adams. The correct citation is: Stainback GA, Lai JH, Pienaar EF, Adams DC, Wiederholt R, Vorseth C (2020) Public preferences for ecological indicators used in Everglades restoration. PLoS ONE 15(6): e0234051. https://doi.org/10.1371/journal.pone.0234051

## References

[1] Stainback GA, Lai JH, Pienaar EF, Adam DC, Wiederholt R, Vorseth C (2020) Public preferences for ecological indicators used in Everglades restoration. PLoS ONE 15(6): e0234051 10.1371/journal.pone.0234051 32555611 PMC7302914

