# Supplementary material for: Public preferences for ecological indicators used in Everglades restoration
Source: PLoS One. 2020 Jun 18;15(6):e0234051. doi: 10.1371/journal.pone.0234051 (PMC7302914; doi:10.1371/journal.pone.0234051)
Supplement: S1 Table — (DOCX) [file pone.0234051.s001.docx]

Table S1 Comparison between the demographics of the survey sample respondents and the Florida population based on 2010 U.S. Census information.

| **Demographic Variable** | **Florida Population** | **Survey Sample** |
| --- | --- | --- |
| Gender (% Female) | 51.10% | 51.10% |
| Household Size | 2.64 | 2.55 |
| Median Income ($) | 48,900 | 70,000 |
| Age | | |
| *Between 18 and 65* | 77.97% | 79.47% |
| *65+* | 22.02% | 19.90% |
| Education | | |
| *Less than High School* | 12.80% | 2.38% |
| *High School* | 59.30% | 45.67% |
| *Bachelor's or Higher* | 27.90% | 51.95% |
| Ethnicity | | |
| *White* | 77.60% | 70.38% |
| *African American* | 16.80% | 17.48% |
| *American Indian or Alaska Native* | 0.50% | 0.29% |
| *Asian* | 2.90% | 3.00% |
| *Pacific Islander* | 0.10% | 0.38% |
| *Other* | 0.00% | 5.10% |
| *Multiple* | 2.10% | 3.38% |
| Hispanic | 24.90% | 27.90% |
| Political Affiliation^1^ | | |
| *Republican (Conservative, Score of 1-3)* | 35.27% (Active Registered Voters) | 41% |
| *Democrat (Liberal, Score of 5-7)* | 37.28% (Active Registered Voters) | 24% |
| *No Affiliation (Neutral, Score of 4)* | 26.89% (Active Registered Voters) | 31% |
| ^1^The comparison of political affiliation should be done with caution as self-reported liberal/conservative political ideology is imperfectly correlated with party affiliation. | | |
