# Supplementary material for: Public preferences for ecological indicators used in Everglades restoration
Source: PLoS One. 2020 Jun 18;15(6):e0234051. doi: 10.1371/journal.pone.0234051 (PMC7302914; doi:10.1371/journal.pone.0234051)
Supplement: S2 Table — (DOCX) [file pone.0234051.s002.docx]

Table S2. Regression results from conditional logit model with willingness to pay Estimates.

|  | **Coefficient** | **Std. Error** | **WTP** |
| --- | --- | --- | --- |
| optout | -2.235*** | 0.072 |  |
| bird10 | 0.475*** | 0.040 | $42.39 |
| bird50 | 0.450*** | 0.038 | $46.46 |
| bird75 | 0.101*** | 0.046 | $44.09 |
| gator10 | 0.087** | 0.042 | $9.93 |
| gator50 | 0.142** | 0.040 | $8.51 |
| gator75 | 0.075*** | 0.047 | $13.95 |
| snail10 | 0.365 | 0.047 | $7.37 |
| snail50 | 0.299*** | 0.037 | $35.73 |
| snail75 | 0.314*** | 0.044 | $29.25 |
| trout10 | 0.301*** | 0.045 | $30.78 |
| trout50 | 0.418*** | 0.036 | $29.50 |
| trout75 | 0.500*** | 0.046 | $40.91 |
| water10 | 0.847*** | 0.044 | $48.91 |
| water50 | 0.475*** | 0.040 | $82.87 |
| water75 | 0.450*** | 0.038 | $95.08 |
| cost | -0.010*** | 0.001 |  |
| Log Likelihood | -13,040.436 |  |  |
| N | 37,797 |  |  |
| LR chi^2^(17) | 1,601.96 |  |  |
| Prob>chi^2^ | 0.0000 |  |  |
| Pseudo R^2^ | 0.0579 |  |  |
| *** 1% significance level ** 5% significance level * 10% significance level | | | |
